# Glucagon-like peptide 1 receptor stimulation reverses key deficits in distinct rodent models of Parkinson's disease

**DOI:** 10.1186/1742-2094-5-19

**Published:** 2008-05-21

**Authors:** Alexander Harkavyi, Amjad Abuirmeileh, Rebecca Lever, Ann E Kingsbury, Christopher S Biggs, Peter S Whitton

**Affiliations:** 1 Department of Pharmacology, The School of Pharmacy, 29-39 Brunswick Square, London WC1N 1AX, UK; 2 Rita Lila Weston Institute of Neurological Studies, 1 Wakefield Street, London WC1N 1PJ, UK; 3 School of Biosciences, University of Westminster, 115 New Cavendish Street, London W1W 6UW, UK

## Abstract

**Background:**

It has recently become apparent that neuroinflammation may play a significant role in Parkinson's disease (PD). This is also the case in animal paradigms of the disease. The potential neuroprotective action of the glucagon-like peptide 1 receptor (GLP-1R) agonist exendin-4 (EX-4), which is protective against cytokine mediated apoptosis and may stimulate neurogenesis, was investigated In paradigms of PD.

**Methods:**

Two rodent 'models' of PD, 6-hydroxydopamine (6-OHDA) and lipopolysaccaride (LPS), were used to test the effects of EX-4. Rats were then investigated *in vivo *and *ex vivo *with a wide range of behavioural, neurochemical and histological tests to measure integrity of the nigrostriatal system.

**Results:**

EX-4 (0.1 and 0.5 μg/kg) was given seven days after intracerebral toxin injection. Seven days later circling behaviour was measured following apomorphine challenge. Circling was significantly lower in rats given EX-4 at both doses compared to animals given 6-OHDA/LPS and vehicle. Consistent with these observations, striatal tissue DA concentrations were markedly higher in 6-OHDA/LPS + EX-4 treated rats versus 6-OHDA/LPS + vehicle groups, whilst assay of L-DOPA production by tyrosine hydroxylase was greatly reduced in the striata of 6-OHDA/LPS + vehicle rats, but this was not the case in rats co-administered EX-4. Furthermore nigral TH staining recorded in 6-OHDA/LPS + vehicle treated animals was markedly lower than in sham-operated or EX-4 treated rats. Finally, EX-4 clearly reversed the loss of extracellular DA in the striata of toxin lesioned freely moving rats.

**Conclusion:**

The apparent ability of EX-4 to arrest progression of, or even reverse nigral lesions once established, suggests that pharmacological manipulation of the GLP-1 receptor system could have substantial therapeutic utility in PD. Critically, in contrast to other peptide agents that have been demonstrated to possess neuroprotective properties in pre-clinical models of PD, EX-4 is in current clinical use in the management of type-II diabetes and freely crosses the blood brain barrier; hence, assessment of the clinical efficacy of EX-4 in patients with PD could be pursued without delay.

## Background

The well-characterised features of Parkinson's disease (PD) are largely the result of a selective degeneration of nigrostriatal neurons, greatly reduced synthetic capacity for dopamine and a consequent failure to engage striatal dopamine receptors [1]. Before the disease presents clinically, death of nigrostriatal neurons occurs in the substantia nigra pars compacta (SNc) 'silently', probably as a result of concurrent apoptotic, excitotoxic, free-radical mediated and neuroinflammatory events [2-5]. Environmental and toxicological factors including bacterial toxins,, in addition to the expression of candidate 'Parkinsonian' genes have all been proposed in the aetiology of the disease [2,3,5]. Despite four decades of research effort, a therapeutic strategy offering a cure for, or a means of arresting the pathology of PD remains elusive. Established drug-based therapies are essentially palliative and not effective in all patients. Moreover, the side effect profiles of most drugs used in PD account for significant morbidity, especially with chronic use, whilst the efficacy of these treatments inevitably diminishes with time as cell loss proceeds [6]. A need exists, therefore, for a novel therapeutic approach, which is both affordable and more importantly, provides the potential for *arresting *disease progression as well as ameliorating symptoms. Since apoptotic cell death is almost certainly one of the central components in selective nigrostriatal neuronal death [7-9] future therapeutic strategies could involve the targeted use of bio-molecules with 'anti-apoptotic' properties. Alternatively, a positive therapeutic effect could be produced by molecules with neurotrophic properties or the ability to stimulate neurogenesis of cells with a dopaminergic phenotype. However, for such an approach to have an improved beneficial effect it would be desirable to stabilize the destructive elements which favour neurodegeneration within the nigra. Compounds with anti-inflammatory properties could be beneficial in this respect. It has recently been observed that the glucagon-like peptide-1 receptor (GLP-1R) agonist exendin-4 (EX-4) shows neurotrophic [10] and 'neuroprotective' [11] properties in cultured PC12 cells subjected to excitotoxic stress. EX-4 – is also able to restore choline acetyl transferase positive cells following ibotenic acid lesions [11]. In addition Perry et al. [12] have found EX-4 to be neuroprotective in a model of neuropathy. These observations have led these authors to suggest that stimulation of GLP-1 receptors (GLP-1R's) could be a therapeutic option in neurodegenerative disorders such as PD. Indeed, EX-4 has been shown to have anti-apoptotic properties against a number of pro-inflammatory mediators in pancreatic β-islet cells [13] which could be seen as indirect support for this suggestion, particularly since neuroinflammation may be a significant causative factor in PD [5]. Very recently it has been observed that EX-4 reverses indicators of NS damage in 6-hydroxydopamine (6-OHDA) lesioned rats when given five weeks after toxin injection [14]. This has been suggested to result from increased neurogenesis originating in the subventricular zone (SVZ) by EX-4 [14]. While clearly an important observation, the relative stability of the lesion at this time following 6-OHDA [15] contrasts with the ongoing hostile environment which prevails in PD patients or at a time point much sooner after neurotoxin administration in animals. Therefore, whether EX-4 mediated recovery would occur under these conditions is as yet unclear. Neuropeptides, such as glial cell derived neurotophic factor, are emerging as potential therapeutic agents in PD [16]. We have recently observed urocortin I (UCN), an endogenous peptide agonist at corticotrophin releasing factor (CRF) receptors with anti-inflammatory properties [17], to be protective in rodent models of PD [18,19]. However, neither GDNF or UCN are able to cross the blood brain barrier meaning that they must either be delivered by neurosurgery or appropriate agonists developed and validated. EX-4 is currently in clinical use in patients with type II diabetes and, despite being a peptide with a molecular weight similar to UCN, is readily able to enter the CNS [20]. Here we have studied the effects of EX-4 on behavioral, neurochemical and histological indices of nigrostriatal damage in both the 6-hydroxydopamine (6-OHDA) or lipopolysaccaride (LPS) lesioned rat. Two distinct models of PD were utilized to diminish the possibility that EX-4 is active against a single unique component of either model. We report here a dramatic and potent reversal by EX-4 of established PD-like lesions in the rat which could be of considerable therapeutic significance if reproduced in the clinic.

## Materials and methods

### Materials

Desmethylimipramine, exendin-4, LPS (serotype E. Coli 0111:B4), pargyline, catalase, L-tyrosine, ferrous ammonium sulphate, benserazide, 6 MPH_4_, 6-OHDA, LPS, apomorphine hydrochloride, 3-iodo-L-tyrosine, D-tyrosine and tyrosine hydroxylase were all obtained from Sigma, UK. Apomorphine and 6-OHDA were dissolved in 2.0% w/v ascorbic acid, whilst UCN solid was initially dissolved in 70% ethanol and further diluted in saline to 1 × 10^-8 ^M stock concentration. All drugs, apart from LPS and 6-OHDA were injected in a volume of 0.1 ml/100 g body weight. Rabbit polyclonal anti-rat TH IgG was obtained from Calbiochem. Horseradish peroxidase (HRP) conjugated goat anti-rabbit IgG was obtained from Cell Signaling, MA. Biotinylated swine anti-rabbit IgG was obtained from Dako, Denmark. ABC complex was purchased from Vector Laboratories, UK. All other reagents were of Analar or HPLC grade.

### Surgical procedures

**Figure 1:**
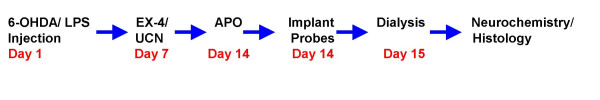
Flow diagram of the protocols used in the present study and the sequence in which they were performed. Groups of rats were put through all of the procedures from toxin injection to *ex vivo *neurochemistry and histology.

An overview of the experimental protocols used in the present series of experiments is shown in figure 1. Note that groups of rats went through all the procedures indicated from initial toxin injection to *ex vivo *neurochemistry and histology. Experiments were carried out in accordance with the Animals (Scientific Procedures) Act UK (1986). Male Wistar rats (210–240 g) were group housed and access to food and water was *ad libitum*. Care was taken to minimize animal usage and typically, tissue was used in several different assay paradigms. Prior to surgery, animals to be lesioned with 6-OHDA received pargyline (50 mg/kg, i.p.) and desmethylimipramine (25 mg/kg, i.p.), in order to maximize the selectivity of the toxin for dopaminergic neurons. Animals were anaesthetized with isofluorane (4% for induction, 1.5% for maintenance), secured in a stereotaxic frame (David Kopf, US) and given injections of 6-OHDA (8 μg/4 μl of saline with 1% ascorbic acid) injected into the right medial forebrain bundle (from bregma in mm; A -4.3, L 1.4 and V 8.2) while LPS (2 μg/2 μl saline) was injected into the substantia nigra pars compacta (SNc; from bregma in mm; A -5.2, L 2.2 and V 8.3). Seven days later rats were administered EX-4 (0.1 or 0.5 μg/kg i.p, twice daily) dissolved in 0.9% saline for a period of seven days.

### Assessment of nigrostriatal lesion severity following apomorphine challenge

All animals received an apomorphine challenge (APO; 0.5 mg/kg sc.) in order to assess lesion severity as previously described [21]. Animals were placed in a circular test arena and following a short period of acclimatization, injected with the dopaminergic agonist. Contraversive turns were noted 20 min. post-injection and recorded over a 120 s observation period. Only complete, 'tight' turns were recorded. We have tested the value of observing rats for longer periods of time (up to 60 min) and recording rotations. Although, obviously, the number of rotations was considerably higher using this protocol the relative difference between groups was virtually identical to sampling for the much shorter time period and the overall methodology was far more time efficient.

### In vivo microdialysis

Later that day rats were implanted with concentric dialysis probes of a construction previously described [22]. Probes were bilaterally implanted into each striatum (from bregma; A +0.2, L 3.0, and ventral 8.2) and the following day perfused with an artificial cerebrospinal fluid (aCSF) solution (composition in mM: 2.5 KCl; 125 NaCl; 1.18 MgCl_2_; 1.26 CaCl_2_) as previously used [22] but without addition of the 5-HT reuptake inhibitor citalopram. Following a 1 hour equilibration period four 30 min 'basal' samples were collected prior to infusion of 100 mM K^+^containing aCSF to evoke DA release. In the latter case the concentration of Na^+ ^was decreased accordingly to maintain osmolarity of the aCSF.

### Tissue dopamine assay

Animals received pargyline (50 mg/kg) 30 min prior to sacrifice. Brains were removed, striata dissected and homogenized in ice-cold phosphate buffer (pH 7.4). All homegenates were split into two equal portions, with one half of each treated with 0.2 M perchloric acid (1:10, w/v) containing ascorbic acid (0.2 μM) and EDTA (0.2 μM), to precipitate cell debris. These were then centrifuged at 9000 × g for 15 min at 4°C, supernatants passed through a syringe filter (10 μm pore size) and whole tissue dopamine levels estimated using HPLC with electrochemical detection [22]. Brain blocs containing nigra were rapidly frozen and retained for immunohistochemistry.

### Ex vivo tyrosine hydroxylase assay

TH activity was measured in the remaining homogenates, using a modification of the method of Naoi et al. [23]. Aliquots were incubated with 200 μM L-tyrosine in a total reaction mixture volume of 100 μl. This consisted of the following components: 100 mM sodium acetate-acetic acid buffer (pH 6.0), 2 mM ferrous ammonium sulphate, 1 mM 6 MPH_4_, 10 μg of catalase and 1 mM benserazide, an inhibitor or aromatic L-amino acid decarboxylase (AADC). 6 MPH_4 _solution was firstly made as 10 mM in 1 M mercaptoethanol. The incubation mixture, except for tyrosine and the pteridin cofactor, was preincubated with homegenates at 37°C for 5 min, and the reaction was initiated by addition of the L-tyrosine and 6 MPH_4_. After incubation at 37°C for 10 min, the reaction was terminated by addition of 100 μl perchloric acid (0.1 M, containing 0.4 mM sodium metabisulphite and 0.1 mM disodium EDTA). The sample was then vortexed and left to stand on ice for 10 min, then centrifuged at 1000 × g for 10 min. The supernatant was diluted to 1 in 1000 with mobile phase, then analyzed using HPLC-ED to measure the amount of L-DOPA. As blank, a similar reaction mixture containing D-tyrosine instead of the L-isomer and 100 μM 3-iodo-L-tyrosine was used [23].

### Immunohistochemistry

Slide-mounted 10 μm cryostat sections from flash-frozen rat brain blocs were removed from the freezer and allowed to equilibrate to room temperature for 30 minutes, prior to post fixation in 4% paraformaldehyde, containing 1% gluteraldehyde for 5 minutes at 0°C. Following rinsing in 0.1 M PBS for 5 minutes, sections were dehydrated through graded alcohols and endogenous peroxidase activity was blocked by incubation in 0.3% H_2_O_2 _in methanol for 10 minutes. The sections were then rehydrated and non-specific immunoreactivity was blocked with 10% swine serum in PBS for 10 minutes. Sections were then incubated in primary antibody (rabbit anti-rat TH IgG) at 1:700 in PBS for 16 hours at 4°C. After rinsing, the sections were incubated sequentially in biotinylated swine anti-rabbit antibody 1:250 in PBS (Dako, Denmark) for 30 minutes at room temperature and ABC complex following the manufacturer's instructions. Immunoreactivity was visualized through incubation in 0.5 mg/ml 3-diaminobenzidine (DAB), containing 0.009% H_2_O_2 _for 2 minutes at room temperature. The sections were counterstained in Harris haematoxylin, dehydrated, cleared and mounted for microscopic examination. Sections were viewed under light microscopy (×40 magnification). Digital images were captured using a Leica DC500 system and the manufacturer's software. For each animal, six successive nigral sections were selected from both treated and contralateral side, taken from a starting point of -5.5 mm relative to bregma, using a total of six rats per group.

### Data handling and analysis

Data obtained from apomorphine challenge, whole tissue DA and TH assay studies were expressed as mean values ± S.E.M. Data were subjected to one way or two way ANOVA to identify overall trends, with a *post hoc *Bonferroni's multiple comparison test used to establish significant differences between the groups. Statistical analysis was performed using a proprietary software package (GraphPad Prism™). Numbers of animals used in experiments are detailed in the figure legends. In all cases, comparisons were made with respect to toxin/vehicle values. Statistical significance was set at p < 0.05.

## Results

**Figure 2:**
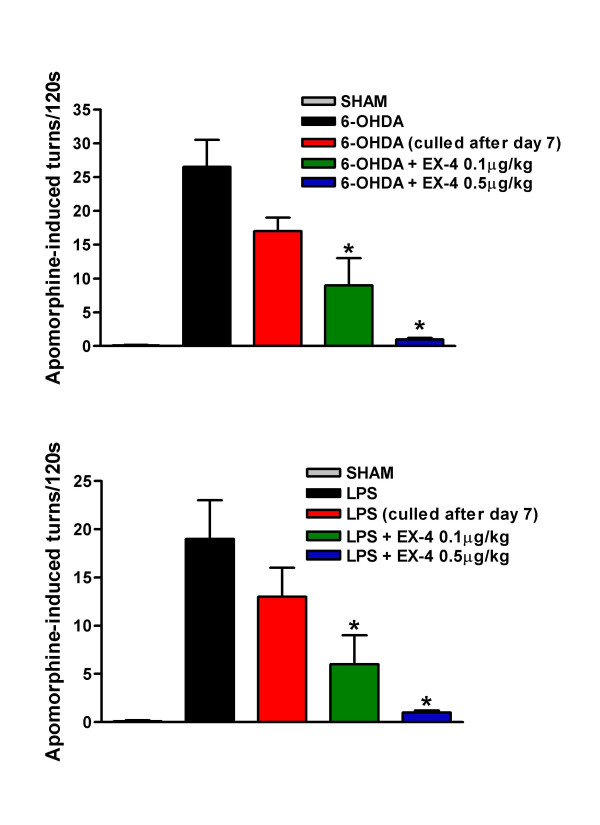
Effect of EX-4 (0.1 and 0.5 μg/kg) on apomorphine-induced rotational behaviour in 6-OHDA (upper panel) or LPS (lower panel) lesioned rats. EX-4 was administered twice daily for seven days seven days *after *toxin injection. Circling was measured for 120 s 30 min after apomorphine injection. One way ANOVA values were 14.90, p < 0.001 (6-OHDA) and 16.24, p < 0.001 (LPS). *indicates significant differences from UCN or sham injection sites (p < 0.01, n = 6 per group).

Apomorphine (APO) induced circling is regarded as a quantitative index of NS lesion severity [21] and thus, an attenuation is predictive of potential anti-parkinsonian activity. Our findings reveal that tight contralateral circling was clearly evident in 6-OHDA and LPS treated rats, but this was dose-dependently attenuated by EX-4 (Fig. 2). Note that lesions are already well-advanced seven days post injection [Fig. 2; [18,19]].

**Figure 3:**
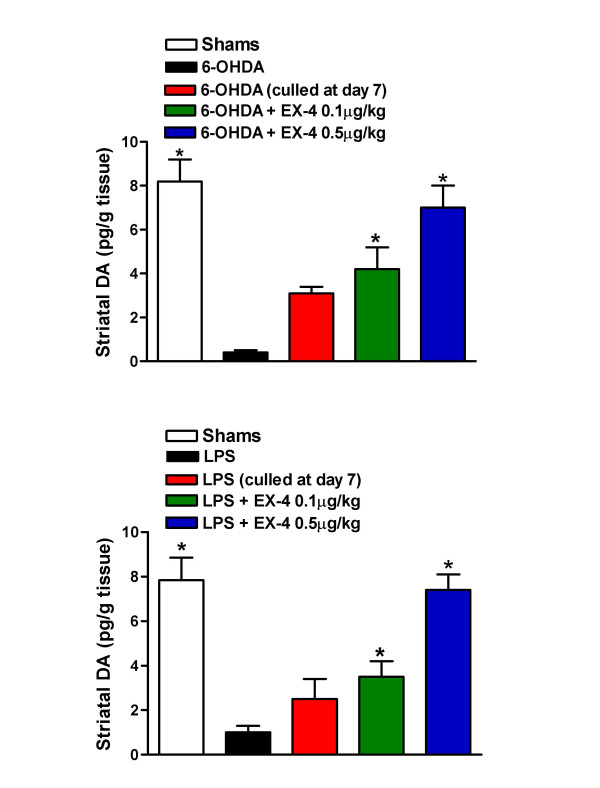
Effect of EX-4 (0.1 and 0.5 μg/kg) on striatal tissue DA content in 6-OHDA (upper panel) or LPS (lower panel) lesioned rats. EX-4 was administered twice daily for seven days seven days *after *toxin injection. One way ANOVA values were 7.88, p < 0.001 (6-OHDA) and 6.67, p < 0.001 (LPS). *indicates significant differences from EX-4 or sham injection sites (p < 0.01, n = 6 per group).

**Figure 4:**
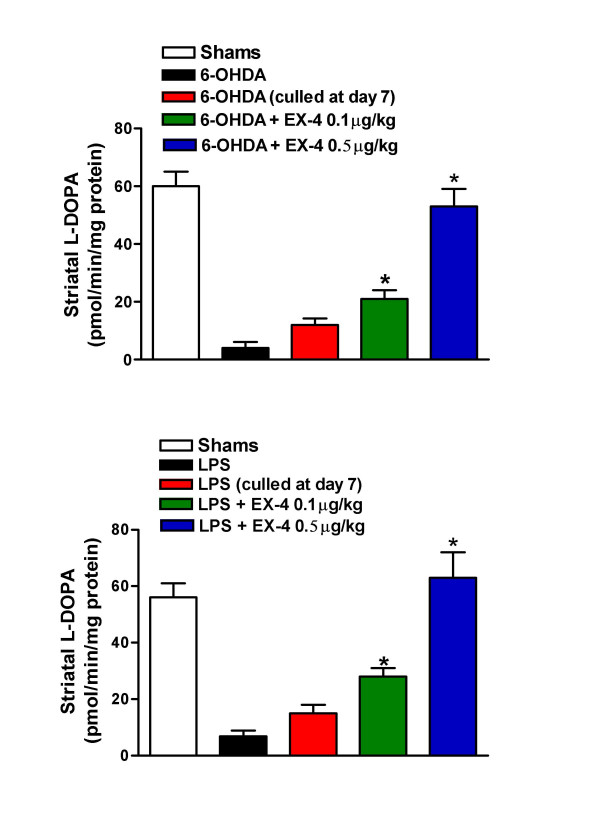
Effect of EX-4 (0.1 and 0.5 μg/kg) on striatal tissue TH activity in 6-OHDA (upper panel) or LPS (lower panel) lesioned rats. EX-4 was administered twice daily for seven days seven days *after *toxin injection. One way ANOVA values were 7.88, p < 0.001 (6-OHDA) and 6.67, p < 0.001 (LPS). *indicates significant differences from EX-4 or sham injection sites (p < 0.01, n = 6 per group).

Striatal tissue DA concentration was substantially reduced in 6-OHDA/LPS treated rats compared with sham or EX-4 groups (Fig. 3). This clearly indicates that EX-4 promotes the retention or restoration of DA cells or cells with a dopaminergic phenotype. In order to estimate functional integrity of nigral neurons ipsilateral to injection sites, we measured L-dihydroxyphenylalanine (L-DOPA) synthetic capacity of striatal homogenates as well as tissue DA content. *Ex vivo *TH activities were significantly reduced in striata of 6-OHDA and LPS treated rats when compared to sham treated rats and this was in marked contrast with animals treated with both EX-4 doses, in which TH activities were near to control values (Fig 4).

**Figure 5:**
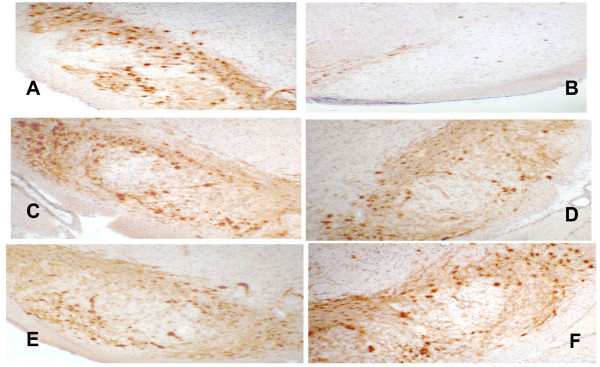
Photomicrographs of selected rat nigral sections, immunostained for TH. Nigrostriatal cell bodies and dendrites appear dark brown/brown in sections. Sections **A, C, E **and are contralateral (untreated) nigra for comparison with ipsilateral (treated) nigra **B, D, F**. Key: **B **– 6-OHDA + vehicle, **D **– 6-OHDA + EX-4 0.1 μg/kg, **F **– 6-OHDA + EX-4 0.5 μg/kg. Bar is 100 μm. Sections are representative of 6 rats for each treatment.

**Figure 6:**
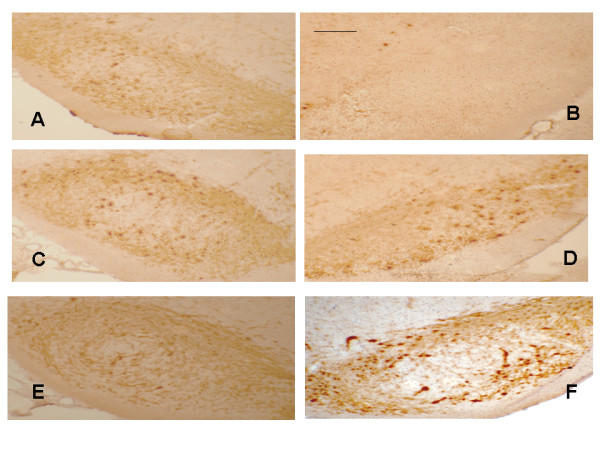
Photomicrographs of selected rat nigral sections, immunostained for TH. Nigrostriatal cell bodies and dendrites appear dark brown/brown in sections. Sections **A, C, E **and are contralateral (untreated) nigra for comparison with ipsilateral (treated) nigra **B, D, F**. Key: **B **– LPS + vehicle, **D **– LPS + EX-4 0.1 μg/kg, **F **– LPS + EX-4 0.5 μg/kg. Bar is 100 μm. Sections are representative of 6 rats for each treatment.

Having established that administration of EX-4 prevents the loss of NS function post-toxin in both rodent models, we needed to ascertain whether this could be explained by preservation of discrete nigral cells or alternatively achieved through a substantial upregulation of TH protein in *surviving *cells. In order to test these possibilities, we stained cryopreserved brain sections for TH immunoreactivity and counted nigral cell bodies in the substantia nigra. As shown in figures 5 and 6, 6-OHDA or LPS treatment resulted in a near complete loss of TH+ cell bodies, with very few accompanying dendrites remaining (Figures show contralateral and ipsilateral nigral slices from the same section). Rats similarly treated, but sacrificed after 7 days show a more modest loss of both NS cell bodies compared to 14 day exposure [18,19]. As can be clearly seen, EX-4 almost completely protected against loss of TH^+ ^cells when administered seven days following 6-OHDA or LPS injection and this was particularly marked in rats given LPS and the higher dose of EX-4. We are unclear as to the reason for this other than to speculate that it may be the result of the toxin used.

**Figure 7:**
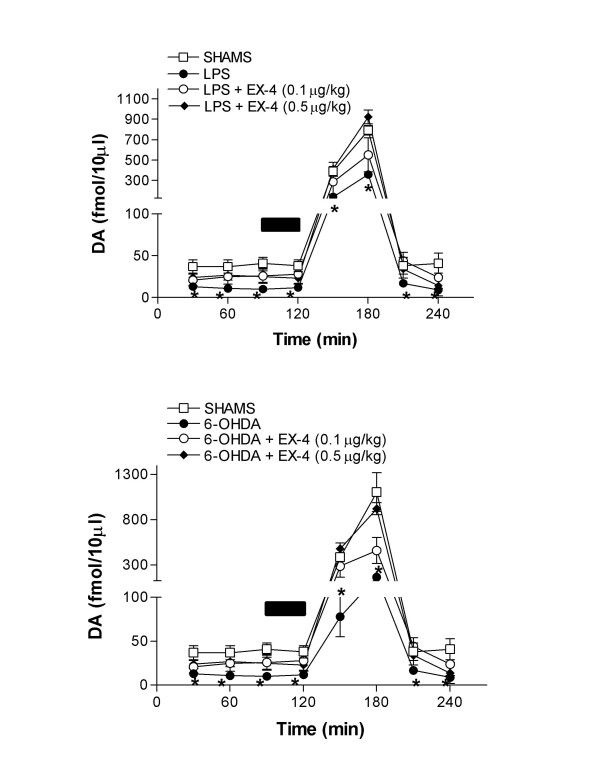
Effect of EX-4 on striatal extracellular DA in 6-OHDA (upper panel) and LPS (lower panel) lesioned rats. The bar indicates the period of 100 mM K^+ ^infusion. Two way ANOVA between treatments were F = 6.16 and 8.77 between treatments and 156.3 and 187.6 over time in 6-OHDA and LPS treated groups respectively. *Denotes differences from sham or EX-4 co-treated rats using Bonferonni's multiple comparison test post hoc. (p < 0.05, n = 6 per group).

In order to evaluate the effect of EX-4 on extracellular DA in the striatum we employed microdialysis in freely moving rats. We have assumed that extracellular DA represents a functional index of DA release and this is supported by our observation that 1 μM tetrodotoxin infusion decreased dialysate DA by over 90% in sham treated rats (data not shown). Both 6-OHDA and LPS greatly reduced extracellular DA (Fig 7), which in a number of samples fell below the limit of detection. In these cases we have assigned a value of 5 fmol/10 μl, which constitutes the level at which an unambiguously identifiable DA peak can be resolved, in order to facilitate analysis of the data. EX-4 dose-dependently reversed the depletion of extracellular DA resulting from either 6-OHDA or LPS (Fig. 7). This restoration of basal and evoked DA release indicates a functional basis upon which motor movement is normalized in apomorphine treated rats (Figs. 2 and 7).

## Discussion

We have used a variety of measures, behavioural, neurochemical and histological which indicate a clear protective role for EX-4 against 6-OHDA or LPS mediated nigrostriatal lesions. These findings support a potential protective role for EX-4 in these two paradigms of PD and clearly demonstrate that when EX-4 is given after either 6-OHDA or LPS, a comprehensive reversal of all selected markers of nigrostriatal cellular loss results. This suggests that EX-4 is able to rescue dopaminergic neurons once damage is established or possibly stimulate neurogenesis of cells with a dopaminergic phenotype. Of particular significance is our finding that EX-4 is able to *arrest and possibly reverse *NS lesions, once the neurodegenerative process has commenced. This principle (if reproduced in the human brain) would have obvious clinical significance since patients generally present with symptoms only once nigral neuronal loss reaches 70–80% of the total nigral compliment, and striatal DA levels have fallen significantly [24]. In the present study, EX-4 appears to 'reverse' loss of TH+ immunoreactivity, since sections taken from rats culled 7 days post-6-OHDA or LPS clearly show greater numbers of TH+ structures than those from rats killed 14 days after toxin injection [18,19].

The underlying cellular mechanism(s) responsible for our finding is as yet, unclear, but other studies [10,11] suggest that increased production of cAMP following GLP-1R stimulation occurs. It has previously been reported that EX-4 has anti-apoptotic properties thereby protecting pancreatic β-cells against pro-inflammatory insults. This may be a significant finding in relation to PD since apoptosis is an important mechanism in 6-OHDA neurotoxicity [25] and human PD [7] and a role for neuroinflammation in both animal models and PD itself is increasingly apparent [4,5]. The use of the single-dose LPS PD model [26] therefore increases the significance of our data. The effects of EX-4 appear to be selectively directed towards the ipsilateral lesioned side as the contralateral side showed no obvious differences in the degree of TH staining between saline and EX-4 treated rats and we have not found any effect of EX-4 on TH staining in naïve rats (data not shown). Moreover, in vitro estimation of DA formation from L-DOPA in naïve rats treated for 7 days with EX-4 were very similar to untreated rats, further indicating a lack of effect of the peptide on TH expression under these conditions (data not shown). It is possible that EX-4 is instigating *de novo *neurogenesis, presumably from recruited stem cells, as has been postulated in other studies [14,27], however, this is clearly speculative and requires futher detailed investigation. The precise neuroanatomical site at which EX-4 acts is therefore as yet unclear and studies, which we are currently undertaking, on the cellular location of GLP1R's in both rodents and patients are needed to shed further light on this.

## Conclusion

Overall, our results significantly add to a growing knowledge base, whereby EX-4 mediates a functional neuroprotection [10-12,14] with the crucial refinement that we have demonstrated, for the first time, efficacy in two distinct *in vivo *models of a currently incurable neuropathology. The apparent ability of EX-4 to arrest NS damage and possibly stimulate remaining cells, suggests a novel mechanism, which if translated therapeutically would offer a significant advance in PD treatment. This principle has recently been proposed as an essential prerequisite for the basis of a meaningful advance in PD therapy [28]. Vitally, EX-4 has a long plasma half life compared with endogenous GLP-1 [20], readily crosses the blood brain barrier and is in current clinical use. This suggests that EX-4 could, in principal, be trialed in PD patients with relatively little delay.

## Abbreviations

DA: dopamine; EX-4: exendin-4; GLP-1: glucagon-like peptide 1; GLP-1R: glucagon-like peptide 1 receptor; LPS: lipopolysaccharide; 6-OHDA: 6-hydroxydopamine; TH: tyrosine hydroxylase; SNc: Substantia nigra pars compacta.

## Competing interests

The authors declare that they have no competing interests.

## Authors' contributions

AA, AH, AK and PSW were responsible for the planning and actual experimentation involved in this study. RL and CSB contributed to the interpretation of the data and writing of the manuscript. The manuscript was read and approved by all of the authors.

## References

[B1] Clark D, White FJ (1987). Review: D1 dopamine receptor B the search for a function: a critical evaluation of the D1/D2 dopamine receptor classification and its functional implications. Synapse.

[B2] Vaux DL, Korsmeyer SJ (1999). Cell death in development. Cell.

[B3] Gandhi S, Wood NW (2005). Molecular pathogenesis of Parkinson's disease. Hum Mol Genet.

[B4] Block ML, Zecca L, Hong J-S (2007). Microglia-mediated neurotoxicity: Uncovering the molecular mechanisms. Nature Neurosci.

[B5] Whitton PS (2007). Inflammation as a potential causative factor in the aetiology of Parkinsons disease. Br J Pharmacol.

[B6] Hurtig HI (1997). Problems with current pharmacological treatment of Parkinson's disease. Exp Neurol.

[B7] Tatton NA, Maclean-Fraser A, Tatton WG, Perl DP, Olanow CW (1998). A fluorescent double-labeling method to detect and confirm apoptotic nuclei in Parkinson's disease. Ann Neurol.

[B8] Ochu EE, Rothwell NJ, Waters CM (1998). Caspases mediate 6-hydroxydopamine induced apoptosis but not necrosis in PC12 cells. J Neurochem.

[B9] Schapira AH (2001). Causes of neuronal death in Parkinson's disease. Adv Neurol.

[B10] Perry T, Lahiri DK, Chen D, Zhou J, Shaw KTY, Egan JM, Grieg NH (2002). A novel neurotrophic property of glucagons-like peptide 1: a promoter of nerve cell growth factor mediated differenciation in PC12 cells. J Pharmacol Exp Ther.

[B11] Perry TA, Haughey NJ, Mattson MP, Egan JM, Grieg NH (2002). Protection and reversal of excitotoxic neuronal damage by glucagon-like peptide-1 and exendin-4. J Pharmacol Exp Ther.

[B12] Perry TA, Holloway HW, Weerasuriya A, Mouton PR, Duffy K, Mattison JA, Grieg NH (2007). Evidence of GLp-1-mediated neuroprotection in an animal model of pyridoxine-induced peripheral sensory neuropathy. Exp Neurol.

[B13] Li L, El-Kholy W, Rhodes CJ, Brubaker PL (2003). Glucagon-like peptide-1 receptor signaling modulates beta cell apoptosis. J Biol Chem.

[B14] Bertilsson G, Patrone C, Zachrisson O, Andersson A, Dannnaeus K, Heidrich J, Kortesmaa J, Mercer A, Neilsen E, Ronnholm H, Wilkstrom L (2008). Peptide hormone exndin-4 stimulates subventricular zone neurogenesis in the adult rodent brain and induces recovery in an animal model of parkinson's disease. J Neurosci Res.

[B15] Zuch CL, Nordstroem VK, Briedrick LA, Hoernig GR, Granholm AC, Bickford PC (2000). Time course of degenerative alterations in nigral dopaminergic neurons following a 6-hydroxydopamine lesion. J Comp Neurol.

[B16] Yasuhara T, Shingo T, Date I (2007). Intracerebral transplantation of genetically engineered cells for Parkinson's disease: toward clinical application. Cell Transplant.

[B17] Gonzalez-Rey E, Fernandez-Martin A, Chomy A, Delgado M (2006). Therapeutic effect of urocortin and adrenomedullin in a murine model of Crohn's disease. Gut.

[B18] Abuirmeileh A, Lever R, Kingsbury AE, Lees AJ, Locke IC, Knight RA, Chowdrey HS, Biggs CS, Whitton PS (2007). The corticotrophin releasing factor-like peptide urocortin reverses key deficits in two rodent models of Parkinson = s disease. Eur J Neurosci.

[B19] Abuirmeileh A, Harkavyi A, Lever R, Biggs CS, Whitton PS (2007). Urocortin, a CRF-like peptide, restores key indicators of damage in a neuroinflammatory model of Parkinson's disease. J Neuroinflammation.

[B20] King AB, Wolfe G, Healy S (2006). Clinical observations of exenatide treatment. Diabetes Care.

[B21] Ungerstedt U (1971). Postsynaptic supersensitivity after 6-hydroxydopamine induced degeneration of the nigro-striatal dopamine system. Acta Physiol Scand Suppl.

[B22] Biggs CS, Fowler LJ, Pearce BR, Whitton PS (1992). Regional effects of sodium valproate on extracellular concentrations of 5-hydroxytryptamine, dopamine and their metabolites in the rat brain: An in vivo microdialysis study. J Neurochem.

[B23] Naoi M, Takahashi T, Nagatsu T (1988). Simple assay procedure for tyrosine hydroxylase activity by high-performance liquid chromatography employing coulometric detection with minimal sample preparation. J Chromatogr.

[B24] Abercrombie ED, Bonatz AE, Zigmond MJ (1990). Effects of L-dopa on extracellular dopamine in striatum of normal and 6-hydroxydopamine-treated rats. Brain Res.

[B25] Cutillas B, Espejo M, Gil J, Ferrer I, Ambrosio S (1999). Caspase inhibition protects nigral neurons against 6-OHDA-induced retrograde degeneration. Neuroreport.

[B26] Herrera AJ, Castano A, Venero JL, Cano J, Machado A (2000). The single intranigral injection of LPS as a new model for studying the selective effects of inflammatory reactions on the dopaminergic system. Neurobiol Dis.

[B27] Borta A, Hoglinger GU (2007). Dopamine and adult neurogenesis. J Neurochem.

[B28] Meissner WT, Hill MP, Tison F, Gross CE, Bezard E (2004). Neuroprotective strategies for Parkinson's disease: conceptual limits of animal models and clinical trials. Trends Pharmacol Sci.

